# Comparison of linear and threshold models for genetic evaluation of morphological defects in Nellore cattle

**DOI:** 10.1093/jas/skaf438

**Published:** 2025-12-24

**Authors:** Milena Aparecida Ferreira Campos, Hinayah Rojas De Oliveira, Henrique Alberto Mulim, Eduarda Da Silva Oliveira, Jorge Hidalgo, Raphael Bermal Costa

**Affiliations:** School of Veterinary Medicine and Animal Science, Federal University of Bahia, Salvador, 40170-110, Brazil; Department of Animal Sciences, Purdue University, West Lafayette, IN 47907; Department of Animal Sciences, Purdue University, West Lafayette, IN 47907; Department of Animal Sciences, Purdue University, West Lafayette, IN 47907; Department of Animal Sciences, Purdue University, West Lafayette, IN 47907; School of Agriculture and Veterinary Science, São Paulo State University, Jaboticabal, 14884-900, Brazil; Department of Animal and Dairy Science, University of Georgia, Athens, GA 30602; School of Veterinary Medicine and Animal Science, Federal University of Bahia, Salvador, 40170-110, Brazil

**Keywords:** binary traits, breeding values, genomic selection, heritability, liability scale, model comparison

## Abstract

Morphological defects in beef cattle can compromise animal welfare and productivity, yet they remain underexplored in genetic evaluations. In this study, we assessed the prevalence and estimated genetic parameters for seven morphological defects in Nellore cattle, including depigmentation, feet and legs malformation, chamfer deviations, loin and jaw defects, hump irregularities, and navel abnormalities, using linear and threshold models. Data from over 180,000 animals recorded between 1998 and 2021 were analyzed. Defect prevalence increased over time, likely due to improved phenotyping and broader participation in data collection. After appropriate scale conversion, linear and threshold models yielded similar heritability estimates; with heritability ranging from 0.03 to 0.12 across traits. Genomic estimated breeding values from both models were expressed on the probability scale, with Spearman correlations of probability scaled values ranging from 0.89 to 0.94 across models. Agreement among commonly selected sires was also high (Spearman 0.94 to 0.97), indicating consistent rankings across models. Feet and legs malformations showed moderate positive correlations with chamfer (0.50) and jaw defects (0.51); chamfer had moderate correlated with hump (0.52); and loin had low positive correlated with jaw (0.34) and with feet and legs (0.39). Depigmentation showed weak correlations with all other traits (<0.20), suggesting distinct genetic control. These results support including morphological defects in routine genetic evaluations and underscore the value of model-appropriate transformations to maintain ranking consistency and interpretability.

## Teaser

This study provides the most comprehensive genetic evaluation of morphological defects in Nellore cattle to date, comparing linear and threshold models across over 180,000 animals. By introducing probability-scaled genomic estimated breeding values (GEBVs), it delivers actionable tools for reducing defect prevalence, improving animal welfare, and guiding breeding programs worldwide.

## Introduction

Despite advances in the genetic improvement of the Nellore breed through selection, morphological defects persist as an issue in production systems. These defects, which include feet and legs malformations, crooked muzzle, deviated hump, jaw abnormalities, and pendulous or very short navel, are not just aesthetic concerns; they have serious implications for the animals’ health, welfare, and productivity (Fernandes Júnior et al., 2022). Affected animals frequently face premature culling, which curtails their productive lifespan and leads to considerable economic losses for producers due to reduced efficiency and increased management demands (Fernandes Júnior et al., 2022; Silva et al., 2023). Furthermore, the welfare of animals with structural deformities is often compromised, as they often experience pain, limited mobility, and reduced fitness, which further compromises their productivity (Souza et al., 2020). Consequently, mitigating the prevalence of these defects through robust genetic selection is of paramount importance, not only for enhancing profitability but also for upholding and improving animal welfare standards across the beef industry.

Incorporating morphological defects into breeding programs requires the definition of optimal statistical models to evaluate these traits, along with the accurate estimation of variance components and key genetic parameters. Estimates of heritability and genetic correlations are particularly crucial, as they provide valuable insights into the inheritance patterns of these complex traits, thereby facilitating their strategic integration into selection programs (Zhu et al., 2023). Therefore, accurate estimation of genetic parameters is an essential first step toward incorporating morphological defects into modern breeding programs, enabling breeders to make informed decisions that balance genetic gain with animal welfare (Ghavi Hossein-Zadeh, 2021; Oliveira Junior et al., 2021). Recent studies have estimated genetic parameters for some morphological defects in cattle. For instance, heritability estimates for navel traits have been reported as 0.33 for Bradford (Souza et al., 2020) and 0.37 for Nellore (da Silveira et al., 2021) cattle, suggesting the potential for selecting genetically superior animals with optimal navel size. Silva et al. (2023) estimated a heritability of 0.18 for feet and legs malformations in Nellore cattle, using a population composed of 295,031 animals. However, to the best of our knowledge, genetic parameters for other morphological defects such as jaw, loin, hump, and chamfer, as well as their current prevalence in the population, remain largely underexplored in cattle.

Morphological defects are usually identified through visual inspection and can be classified as threshold traits due to their categorical nature (e.g., scores) or binary distribution (Falconer and Mackay, 1996). In many practical scenarios, these morphological defects are recorded as binary traits (present or absent), and they generally tend to exhibit reduced environmental influence (Rosa et al., 2013). Threshold models are considered the most suitable statistical approach for the analysis of binary and/or categorical traits (Zhou et al., 2018). These models assume that an underlying liability follows a normal distribution and that the observed phenotype (i.e., presence or absence of a defect) is dictated by the liability exceeding or not a fixed threshold (Harville and Mee, 1984). Importantly, breeding values and heritability estimates from threshold models are expressed on the liability scale, which cannot be interpreted directly and therefore require transformation to the probability scale (e.g., via inverse probit or logit) for practical interpretation.

Despite their theoretical appeal, threshold models rely on complex, often iterative, algorithms for the estimation of genetic parameters. This complexity renders them computationally intensive, especially for large datasets (Hidalgo et al., 2024a). Consequently, threshold models may encounter issues with convergence and estimation reliability (Hidalgo et al., 2024a). Linear models, in contrast, provide a more computationally efficient alternative, even though they were originally designed for continuous traits (Zhou et al., 2018). When applied to binary data, linear models treat the outcome as if their residuals had homogeneous variance and followed a normal distribution. Although these assumptions are not fully met for binary traits, linear models often produce estimates of genetic parameters that are highly correlated (often ≥0.96) with those derived from threshold models, particularly after appropriate transformation to the observed scale (Ramirez-Valverde et al., 2001; Koeck et al., 2010). However, linear models generate estimates on the observed scale, which are not bounded between 0 and 1 and therefore do not directly represent probabilities. This limits their intuitive interpretation when the goal is to express genetic merit as the likelihood of defect expression (e.g., Ramirez-Valverde et al., 2001; Cappelloni et al., 2022; Hidalgo et al., 2024b).

To address this limitation, Hidalgo et al. (2024a, 2024b) recently introduced a transformation method that converts genomic estimated breeding values (GEBVs) from linear models to be converted from the observed to the liability scale, thereby enabling interpretation in terms of probabilities. This method was successfully applied to calving ease and health traits in beef (Hidalgo et al., 2024a) and dairy (Hidalgo et al., 2024b) cattle, particularly when the prevalence of the trait was greater than ∼5%. For low-prevalence traits (<5%), a new approach has been recently proposed by de Oliveira Padilha et al. (2025), which was tested and recommended for health traits in dairy cattle. The development of these transformations has important implications for the genetic evaluation of threshold traits, as it allows for the computational efficiency of linear models while maintaining the interpretability of results on the probability scale. However, despite their promising applications, the efficacy and applicability of these transformation methods across a broader range of traits and populations, including morphological defects in Nellore cattle, warrant further investigation. Therefore, the objectives of this study were to: 1) examine the current prevalence and temporal trends of key morphological defects in a large Nellore cattle population; 2) estimate and compare genetic parameters obtained using linear and threshold models for these morphological defects; 3) assess the feasibility of expressing GEBVs for morphological defects in Nellore cattle as probabilities, using both Hidalgo et al. (2024a, 2024b), and de Oliveira Padilha et al. (2025) approaches; and 4) estimate a proxy of genetic correlation among all morphological defects.

## Materials and Methods

The data used in this study originated from the DeltaGen breeding program and were provided by the Gensys company (Porto Alegre, Rio Grande do Sul, Brazil). The DeltaGen program performs the routine genetic evaluation for Nellore animals raised in Brazil. Therefore, animal care and use committee approvals were not required, as the data were sourced from preexisting databases.

### Phenotypic data

Phenotypic data were collected by trained technicians within the DeltaGen program across three distinct evaluation phases: at weaning (approximately 7 months old), yearling (approximately 16 months old), and at the final evaluation stage (approximately 18 months of age). These data collection efforts spanned from 1998 to 2021. During these evaluations, comprehensive records were gathered on growth traits, reproductive performance, and visual assessments of conformation, morphological defects, precocity, and muscularity.

The visual assessment of morphological defects used a binary approach (0 for absence and 1 for presence) to diagnose issues related to feet and legs, chamfer, hump, depigmentation, loin, jaw, and navel. To ensure consistency, all defects were classified following the standardized protocol adopted by the Gensys Company. In this context, feet and legs malformations referred to visible deviations in limb alignment or hoof structure that could impair locomotion. Chamfer defects corresponded to lateral deviation or asymmetry of the muzzle or nasal bone. Hump defects were defined as displacement or irregular conformation of the thoracic hump, typically involving lateral deviation. Loin defects were characterized by irregularities such as depression or asymmetry in the lumbar region. Jaw defects included mandibular abnormalities such as prognathism, brachygnathism, or lateral deviation. Depigmentation referred to partial or complete loss of pigmentation around the muzzle, in the periocular region, on localized body patches, or in the tip of the tail. Navel defects were identified based on the presence of an enlarged, elongated, or pendulous umbilical structure, or by abnormally short navels that exposed the penis in males. The classification criteria used for the morphological defects identified in this population are summarized in the Supplementary Material S1.

The initial raw dataset contained information from 799,672 animals, including individuals with and without records for the defects of interest in this study. Contemporary groups (CG) were defined based on year and season of birth, sex, farm of rearing at weaning and yearling, management group at weaning and yearling, and date of measurement at weaning and yearling. All components included in the CG were tested for statistical significance and retained only when relevant, as demonstrated in previous studies using a similar dataset (Vargas et al., 2022; Silva et al., 2023). In addition, morphological defects in the DeltaGen program are recorded only once per animal at the stage when the defect is first observed (weaning, yearling, or final evaluation), which is already accounted in the CG. Therefore, in this study, CG was the only systematic effect included in the statistical models because it already incorporated all relevant systematic sources of variation (i.e., sex, year and season of birth, farm, management group and date of measurement).

Contemporary groups containing fewer than 10 animals or those exhibiting no phenotypic variability for the trait under analysis were excluded to ensure robustness of estimates. Connectedness among all CGs was verified using the AMC software (Roso and Schenkel, 2006), and any disconnected groups were subsequently removed from the dataset. The complete table with the number of contemporary groups excluded under each criterion is provided in the Supplementary Material S2. The total number of animals after the phenotypic quality control, the number of affected animals, and the number of contemporary groups for each trait are shown in Table 1.

**Table 1.** Total number of animals, number of animals with defects, and number of contemporary groups for each trait after quality control

Trait	Number of animals	Number of animals with defects	Number of contemporary groups
**Depigmentation**	182,964	11,310	5,629
**Feet and legs**	108,782	8,098	3,534
**Chamfer**	118,354	5,972	3,628
**Loin**	81,818	3,894	2,488
**Hump**	60,188	3,363	1,857
**Jaw**	38,105	1,613	1,201
**Navel**	13,745	665	456

### Pedigree and genomic information

The complete pedigree file used in this study contained information for 1,192,464 animals, which included 7,203 sires and 387,261 dams, spanning up to 12 generations. For the analyses, only 3 generations of pedigree were considered. Genomic data were available for 24,729 animals with recorded defects and/or their relatives. The animals were genotyped using Neogen’s 50K SNP chip (GGP Indicus; Neogen 2021) and imputed to a 777K density using the Illumina Bovine HD array (Illumina Inc. 2010), based on a reference population of 6.105 Nellore animals. Imputation was performed as part of Gensys’ official evaluation using the FImpute V3 software (Sargolzaei et al., 2014). The imputation used the same Nellore reference population described by Neves et al. (2014), for which accuracy estimates above 0.97 have been reported. Genotypic quality control (QC) was performed after imputation using the preGSf90 software (Aguilar et al., 2014), following the parameters suggested by Silva et al. (2023). In summary, SNP markers with a *P*-value < 10^−15^ in the Hardy-Weinberg equilibrium test, call rate < 0.90, and minor allele frequency (MAF) < 0.02 were removed from the dataset. After QC, a total of 24,562 animals and 583,769 SNP markers were retained for further analysis.

### Variance components estimation

Variance components and GEBVs were obtained using Bayesian inference, implemented in the GIBBSF90+ software, part of the BLUPF90 family (Misztal et al., 2018). This software facilitates the use of Gibbs sampling, within a Markov Chain Monte Carlo (MCMC) framework to estimate the posterior marginal distributions of the genetic parameters. Each morphological defect was analyzed using two distinct statistical models: a linear and a threshold model, to enable the comparison of their estimates. The single-trait linear and threshold models used in this study can be defined, respectively, as follows:


(1) $$\begin{array}{l} y=X\beta +Za+e\;\text{and}\\ \;l=X\beta +Za+e \end{array}$$

where **y** and **l** are the vectors of records (for the trait or liability scale, respectively); **β** is the vector of systematic effects of contemporary groups, assumed as **β|**$\sigma_{b}^{2}$ ∼ N(0, ${I\sigma }_{b}^{2}$), where $\sigma_{b}^{2}$ has large variances (10^10^) to represent vague prior knowledge; **a** is the vector of direct additive genetic effects, assumed as **a|**$\sigma_{a}^{2},H$ ∼ N(0, $H\sigma_{a}^{2}$), where **H** is the relationship matrix that combines pedigree (**A**; considering up to 3 generations in this study) and genomic (**G**) relationship matrices, and $\sigma_{a}^{2}$ is the direct additive genetic variance. The e is the vector of residual effects, assumed as **e|**$\sigma_{e}^{2}$ ∼ N(0, $I\sigma_{e}^{2}$), where $\sigma_{e}^{2}$ is the residual variance, and **I** is an identity matrix. For the threshold model, the residual variance was fixed to a unity (1). The **X** and **Z** are incidence matrices relating the records to the vectors **β** and **a**, respectively. In the linear model, the observed binary phenotypes are directly fitted into the statistical model. In contrast, for the threshold model, an underlying distribution was assumed as follows:


(2) $$f(y|l,t)=\;\prod_{i=1}^{n}\left[I(l_{i}\leq t)I(y_{i}=1)+I(l_{i}>t)I(y_{i}=2)\right]$$

where **y** is the vector of binary records (1 or 2), $l_{i}$ is the underlying liability of record *i*; t is the threshold that defines the category response for the traits, n corresponds to the number of observations, and I(⋅) is an indicator function taking the value of 1 when the specified condition is true, and 0 otherwise.

The inverse of the hybrid pedigree-genomic relationship matrix (**H^−1^**), was created as described by (Aguilar et al., 2010):


(3) $$H^{-1}=\;A^{-1}+\;[\begin{array}{cc} 0 & 0\\ 0 & G^{-1}-\;A_{22}^{-1} \end{array}]$$

where $A_{22}^{-1}$ is the inverse of the pedigree relationship matrix for the genotyped animals and $G^{-1}$ is the inverse of the genomic relationship matrix, which was constructed as described by VanRaden (2008):


(4) $$G\;=\;\frac{ZZ^{'}}{2\sum_{i=1}^{m}p_{i}(1-p_{i})}$$

where **Z = (M − P)**, in which **M** is the gene content matrix, with *m* columns (number of SNP markers) and *n* lines (number of genotyped animals). The elements in **M** were set to 0, 1, and 2 for the genotypes AA, AB, and BB, respectively. **P** is the matrix with twice the allele frequencies, i.e., 2 pi, and p_i_ is the frequency of the *i*^th^ SNP marker.

The MCMC chains were run for a total of 300K to 1M iterations, with a burn-in period ranging from 30K to 500K, as these parameters ensured the convergence of all chains across all traits and models. A 50-sample thinning interval was used in all analyses to reduce autocorrelation between successive samples. Convergence was assessed by visual inspection, along with the Heidelberger and Welch (1983) and Geweke et al. (1992) criteria, all implemented in the “boa” package (Smith, 2007) available in the R software (R Core Team, 2016). Variance components and genetic parameters were then obtained as the mean of the estimated marginal posterior distributions. To allow a fair comparison of computational performance across models and traits, we summarized both the raw wall-clock runtime and iteration-adjusted computational time. Because traits differed in the number of MCMC iterations required for convergence, runtime was standardized as hours per 100,000 iterations for variance component estimation, and hours per 10,000 iterations for GEBV estimation (Supplementary Tables S3 and S4).

### Model comparison

To make a fair comparison between the variance components estimated from both models, the transformation from the liability scale to the observed scale was performed using the formula proposed by Dempster and Lerner (1950), i.e.:


(5) $$h_{o}^{2}=\frac{z^{2}h_{l}^{2}}{\alpha (1\;-\alpha )}$$

where, $h_{o}^{2}$ is the heritability estimate on the observed scale, z is the height (probability density) of the ordinate of the standard normal probability density function at the point corresponding to the threshold between categories, estimated from the prevalence (α) of traits, and $h_{l}^{2}$ is the heritability estimate on the liability scale.

#### GEBV transformation to probabilities

The GEBVs from both models were converted to the probability scale, as this format is considered the gold standard for binary traits in breeding programs due to its intuitive and practical interpretability. First, GEBVs derived from the threshold model (on the liability scale) were directly transformed to the probability of an animal expressing the defect using the standard normal cumulative distribution function (Φ), as detailed by Hidalgo et al. (2024b):


(6) $$P_{i}\;=\;\Phi \;(\frac{t\;-\;\mu_{U}\;-\;U_{i}}{\sigma_{e}})\;$$

where P*i* is the probability of the animal presenting the defect, Φ is the standard cumulative distribution function, t is the threshold, $\mu_{U}$ is the mean of GEBV, $U_{i}$ is the GEBV for animal i, and $\sigma_{e}$ is the residual SD on the liability scale (typically 1).

For the linear model, GEBVs were initially on the observed scale. Thereafter, two alternative procedures were evaluated to approximate the GEBVs to the liability scale before applying the same probability transformation above (equation 6):

1. Variance-heritability adjustment-scaling based on residual variance and the ratio of heritabilities between the observed and liability scales, following the approach reported in Hidalgo et al. (2024b):
  (7) $${BV}_{l}\approx \;\frac{BV_{o}}{\sqrt{{\sigma_{e}^{2}}_{o}}\times (1-\;\frac{h_{o}^{2}}{h_{l}^{2}})}\;$$
  where BV_l_ and BV_o_ are the estimated breeding values on the liability and observed scales, respectively. The ${\sigma_{e}^{2}}_{o}$ is the residual variance on the observed scale, $h_{o}^{2}$ is the heritability estimate on the observed scale, and $h_{l}^{2}$ is the heritability estimate on the liability scale.
2. Threshold-density adjustment-scaling based on the height of the ordinate of the standard normal distribution at the threshold, derived from the prevalence of the defect in the population, as proposed by de Oliveira Padilha et al. (2025) and conceptually rooted in Dempster and Lerner (1950) and Sorensen and Gianola (2002). In this second approach, the scaling factor (z) is computed as:
  (8) $$z=\sqrt{\frac{h_{o}^{2}}{h_{l}^{2}}\alpha (1-\alpha )}\;$$

where $h_{o}^{2}$ is the heritability estimate on the observed scale, $h_{l}^{2}$ is the heritability estimate on the liability scale, and α is the trait prevalence. The liability scale GEBV is then obtained as:


(9) GEBVl≈GEBVoz#(9)

where all terms were previously defined.

Spearman rank correlations between the probability-scaled GEBVs derived from the threshold model and those obtained from the linear model (via two-step transformation) were calculated for each trait to assess the concordance in animal ranking across the two modeling approaches. These correlations were computed using the *cor()* function in R (R Development Core Team, 2016), based on the method “spearman.”

#### Concordance of top-ranked animals between models

After testing the two approaches of converting GEBVs, the method that produced the highest correlations between linear and threshold models was selected for evaluating the consistency of selection decisions. This step aimed to assess how model choice (linear vs threshold) influences sire ranking particularly in traits with varying prevalence and heritability. Only sires with at least 10 phenotyped offspring per trait were included in this validation (the total number of sires for each trait is included in the Supplementary Material S5). From this reference group, the top 10% sires were selected according to their GEBVs estimated based on each model and scale (observed and liability), potentially resulting in different sets of selected animals.

To assess consistency, we compared the overlap in selected sires across models. We categorized them into four groups: 1) selected by both models, 2) selected only by the linear model, 3) selected only by the threshold model, and 4) not selected by either model. Two types of comparisons were performed: 1) selection overlap between linear models in observed vs. liability scales, and 2) selection overlap between the linear model (after transformation to liability scale) and the threshold model.

### Genetic correlations among morphological defects

Understanding the genetic relationships among morphological defects is essential for an accurate breeding program design and the development of effective selection indices. However, due to convergence issues in multiple-trait models using only morphological defect traits, and limited access to raw phenotypic data for other traits, direct estimation of genetic correlations was not feasible. As an alternative, we approximated genetic correlations by computing Pearson correlation coefficients among GEBVs for each pair of traits.

These correlations were calculated using the same set of sires described above (in the *“Spearman Rank Correlations and Proportion of Commonly Selected Animals”* topic), providing a consistent reference population. GEBVs were derived from each model (linear and threshold) and scale (observed and liability), allowing us to assess how trait relationships may differ depending on the modeling approach. Results offer insights into the degree of shared genetic control among defects and may help prioritize traits for joint selection or inform strategies for minimizing correlated responses.

## Results

### Prevalence of morphological defects

**Figure 1.**
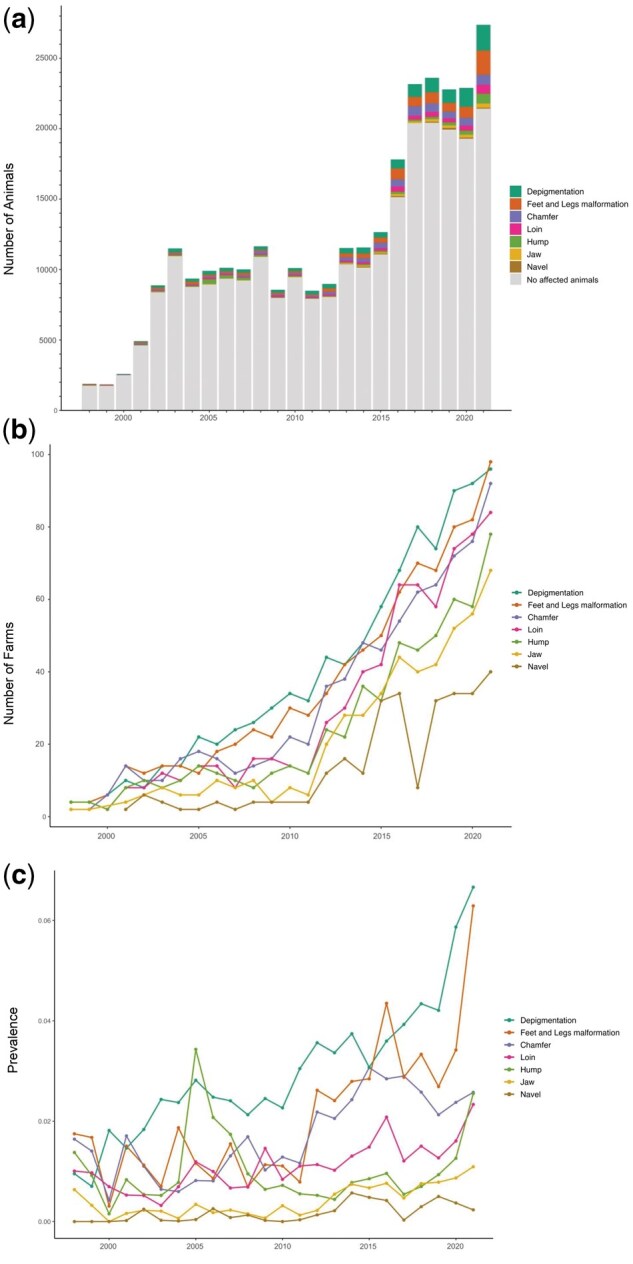
Descriptive analysis of animals and the number of farms evaluated per year. (a) The gray bars represent the total number of animals evaluated by birth year. Colored segments within each bar represent the number of animals with specific morphological defects. (b) Number of farms reporting each defect. (c) Prevalence (in percentage) of the defects over the years.

Temporal trends in the prevalence of these defects from 1998 to 2021 are shown in Figure 1. The overall prevalence were 6.18% for depigmentation, 7.44% for feet and leg malformations, 5.05% for chamfer defects, 4.76% for loin defects, 5.59% for hump defects, 4.23% for jaw defects, and 4.84% for navel abnormalities. Depigmentation (*n* = 93 farms), feet and leg malformations (*n* = 84 farms), and chamfer defects (*n* = 92 farms) were recorded on a high number of farms. Conversely, loin abnormalities (*n* = 74 farms), hump defects (*n* = 73 farms), jaw defects (*n* = 61 farms), and navel (*n* = 47 farms) were less frequently reported. Nevertheless, the number of farms recording all defects substantially increased over the years (Figure 1b).

Although the absolute number of affected animals increased over time, this rise parallels a substantial expansion in the total number of animals phenotypically recorded in the DeltaGen breeding program (Figure 1a), highlighting the need to consider population size (i.e., prevalence and/or proportion of affected animals) when interpreting prevalence trends. In this context, for our Nellore population, prevalence substantially increased over the years for most defects (Figure 1c).

### Model comparison

Genetic parameters, specifically heritabilities, were estimated using both linear and threshold models. The linear mixed model estimates heritability on the observed scale, whereas the threshold model provides estimates on the underlying liability scale. To facilitate the comparison among models, heritability estimates from the threshold model were also transformed to the observed scale using the method described by Dempster and Lerner (1950). Table 2 shows the heritability estimates derived from both models, before and after the transformation to the observed scale. Variance components estimated using the linear and threshold models for each morphological defect are included in the Supplementary Material S6.

**Table 2.** Heritabilities estimated using the threshold (in the liability and observed scale) and linear (observed scale) models. The $h_{o}^{2}$ estimates and their 95% highest posterior density (HPD) intervals shown in the third and fourth columns were obtained by converting values from the liability to the observed scale

	Threshold			Linear		
	$h_{l}^{2}$	HPD interval (95%)	$h_{o}^{2}$	HPD interval (95%)	$h_{o}^{2}$	HPD interval (95%)
**Depigmentation**	0.54	0.51–0.57	0.12	0.11–0.13	0.11	0.10–0.12
**Feet and legs malformation**	0.23	0.20–0.26	0.06	0.04–0.05	0.04	0.03–0.05
**Chamfer**	0.18	0.15–0.21	0.04	0.03–0.04	0.03	0.02–0.03
**Loin**	0.37	0.33–0.42	0.07	0.06–0.08	0.06	0.05–0.07
**Hump**	0.35	0.30–0.41	0.08	0.06–0.09	0.06	0.06–0.07
**Jaw**	0.26	0.20–0.32	0.05	0.04–0.06	0.04	0.03–0.05
**Navel**	0.50	0.39–0.61	0.10	0.08–0.12	0.08	0.05–0.11

$h_{l}^{2}$ : heritability on the liability scale; $h_{o}^{2}$: heritability on the observed scale.

Heritability estimates obtained with the linear model were consistently lower across all traits compared to those from the threshold model when expressed on the liability scale. After converting the estimates generated by the threshold model to the observed scale, the estimates were generally consistent between the two models. For instance, depigmentation and navel had the highest heritabilities (0.12 and 0.10 for the threshold models after conversion to the observed scale, respectively), followed by hump (0.08), loin (0.07), and feet and legs malformation (0.06). Chamfer (0.04) and jaw (0.05) defects had the lowest heritabilities estimated in this study (Table 2). Posterior means of estimates from linear and threshold models are shown in the Supplementary Materials S7 and S8.

**Figure 2.**
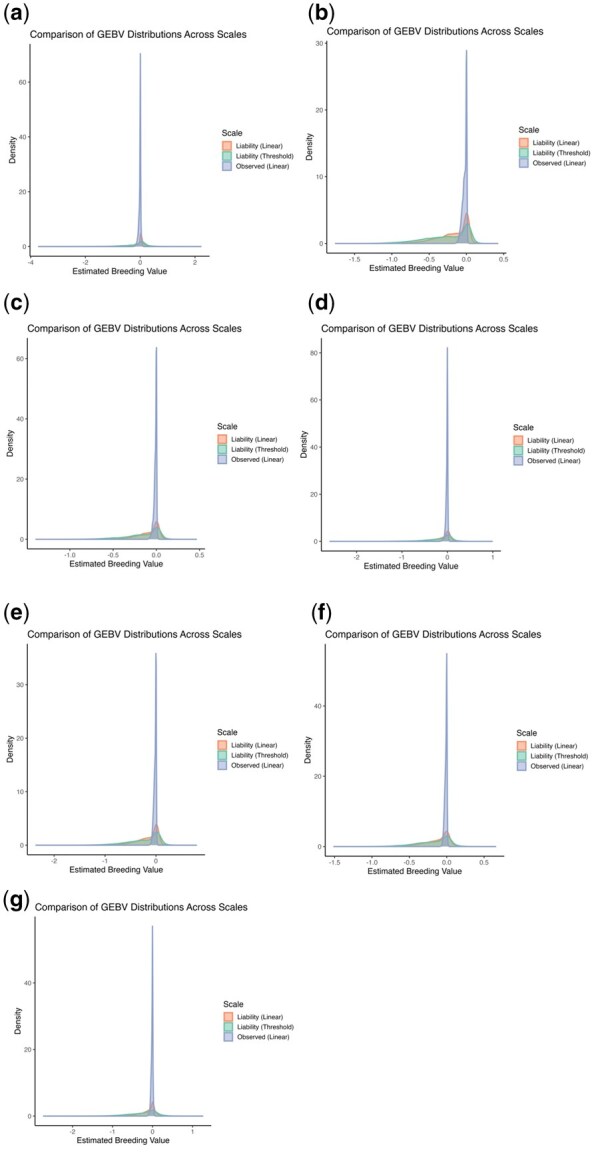
Distribution of GEBVs across different scales. GEBVs obtained from linear models are presented on the observed scale and after approximation to the liability scale, whereas GEBVs obtained from threshold models are presented directly on the liability scale. Traits shown are: (a) Depigmentation, (b) Feet and legs malformations, (c) Chamfer, (d) Loin, (e) Hump, (f) Jaw, and (g) Navel.

Regarding the approximation of the GEBVs from the observed scale (linear model) to liability scale, and for converting GEBVs from both models to the probability scale, the second approach (i.e., Threshold-density adjustment) provided the highest correlations and greatest consistency across all traits (Supplementary Materials S9 and S10). Consequently, GEBVs transformed using the equations from this second approach were used in subsequent analyses. Figure 2 shows the distribution of GEBVs estimated using the linear model (in both the original observed scale and after conversion to the liability scale) and the threshold model (liability scale). If the transformation from the observed to the liability scale is effective, the distributions from the linear and threshold models are expected to overlap closely.

#### Transformation of GEBVs to probabilities

**Figure 3.**
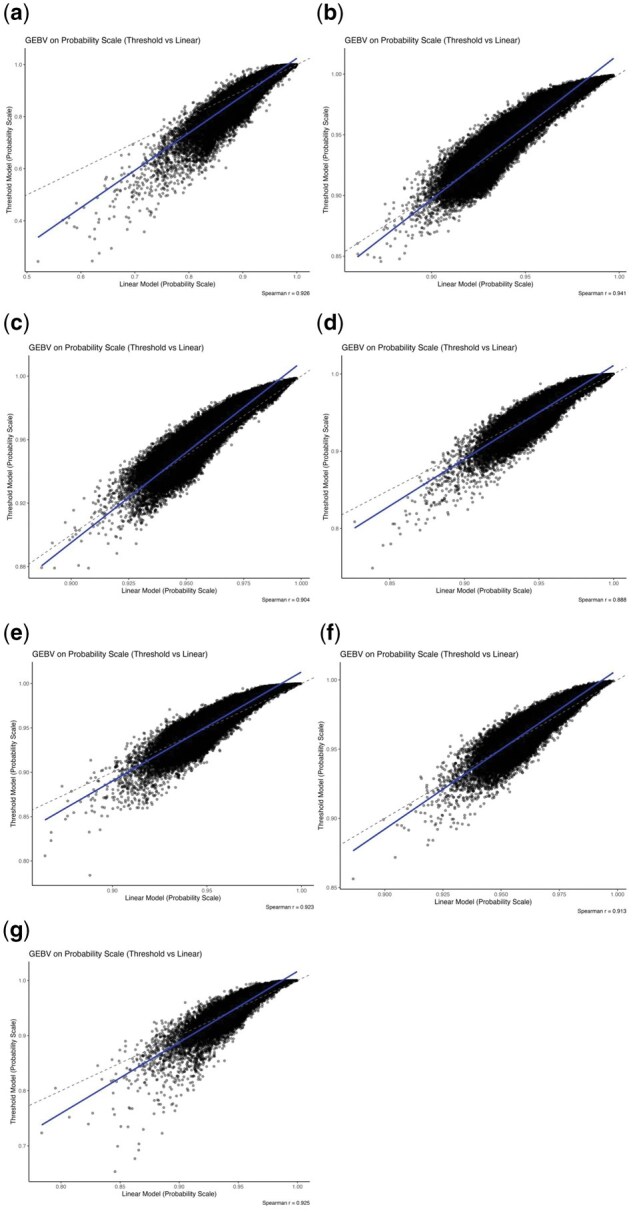
Dispersion plots of probability-scaled GEBVs derived from both linear and threshold models for (a) Depigmentation, (b) Feet and legs malformations, (c) Chamfer, (d) Loin, (e) Hump, (f) Jaw, and (g) Navel.

Spearman’s rank correlation between the probability-scaled GEBVs derived from both linear and threshold models were used to assess changes in ranking for the different defects. Correlation coefficients were high (>0.88) for all traits, indicating strong agreement between models. The dispersion plots of probability-scaled GEBVs from both linear and threshold models are shown in Figure 3, while the dispersion plots of liability scale from both models are shown in the Supplementary Material S11.

#### Concordance of top-ranked animals between models

To evaluate consistency in sire ranking across models, we assessed the overlap in the top 10% selected sires and the corresponding Spearman correlations. The main results for each defect are summarized below.

##### Depigmentation

**Figure 4.**
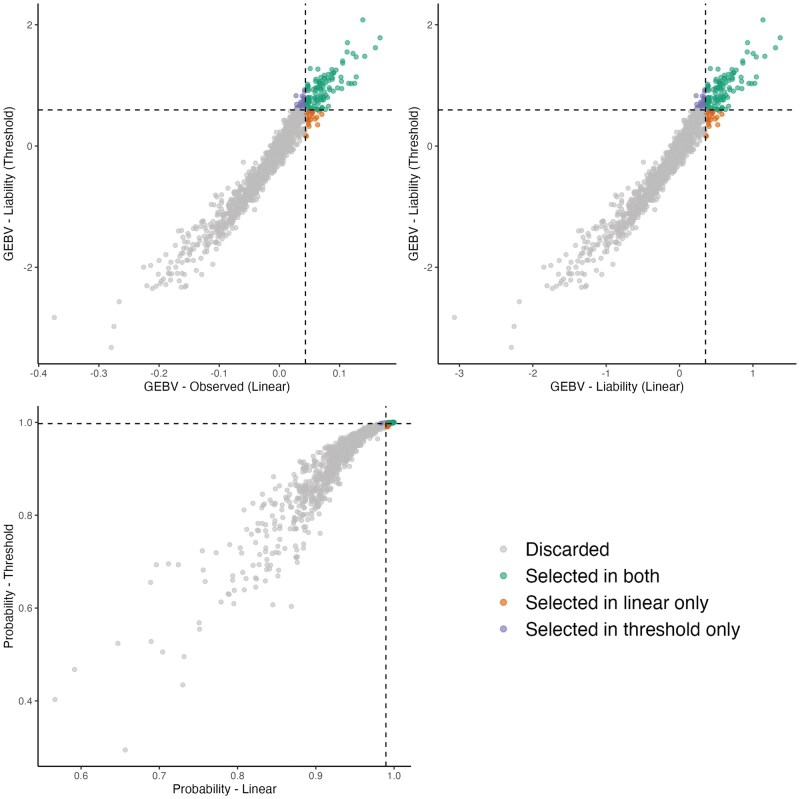
Commonly selected animals for the depigmentation trait across models. Left: GEBVs from the linear model on the observed scale vs. GEBVs from the threshold model on the liability scale, considering only animals with more than 10 offspring. Right: GEBVs from the linear model on the liability scale vs. GEBVs from the threshold model on the liability scale. Bottom: GEBVs from the linear model on the probability scale vs. GEBVs from the threshold model on the probability scale. Animals are classified according to their selection status: not selected in either model, selected in both models, selected only in the linear model, or selected only in the threshold model.

A total of 1,338 sires with more than 10 phenotyped offspring were evaluated, with an average of 130 offspring per sire. Among the top 10% based on GEBVs from the linear and threshold models (134 animals), 106 sires were commonly selected by both models, while 28 were selected exclusively by one or the other. Based on the 10% selection threshold, 1,176 sires were discarded. The Spearman correlation among the top 10% animals was 0.97 for all the scales (Figure 4). The strong correlation between GEBVs in the linear model (observed vs. liability scale) indicate minimal re-ranking due to scale transformation.

##### Feet and legs malformations

**Figure 5.**
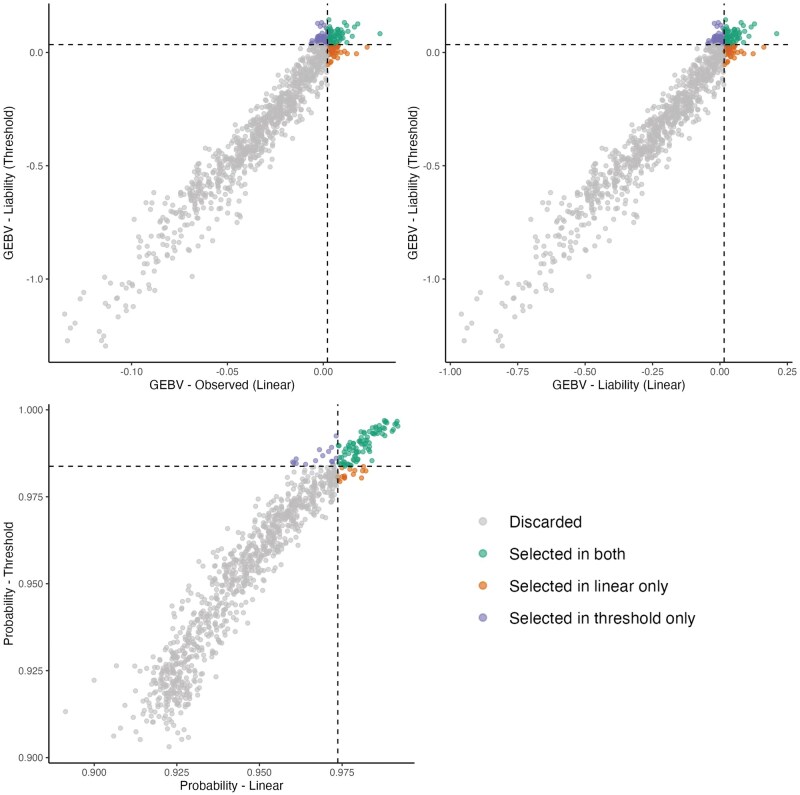
Commonly selected animals for the feet and legs malformations across models. Left: GEBVs from the linear model on the observed scale vs. GEBVs from the threshold model on the liability scale, considering only animals with more than 10 offspring. Right: GEBVs from the linear model on the liability scale vs. GEBVs from the threshold model on the liability scale. Animals are classified according to their selection status: not selected in either model, selected in both models, selected only in the linear model, or selected only in the threshold model.

A total of 987 sires with more than 10 phenotyped offspring were evaluated, with an average of 102 offspring per sire. Among the top 10% based on GEBVs from the linear and threshold models (99 animals), 24 sires were commonly selected by both models, while 75 were selected exclusively by one or the other. Based on the 10% selection threshold, 813 sires were discarded. The Spearman correlation among the top 10% animals was 0.94 for all the scales (Figure 5).

##### Chamfer

**Figure 6.**
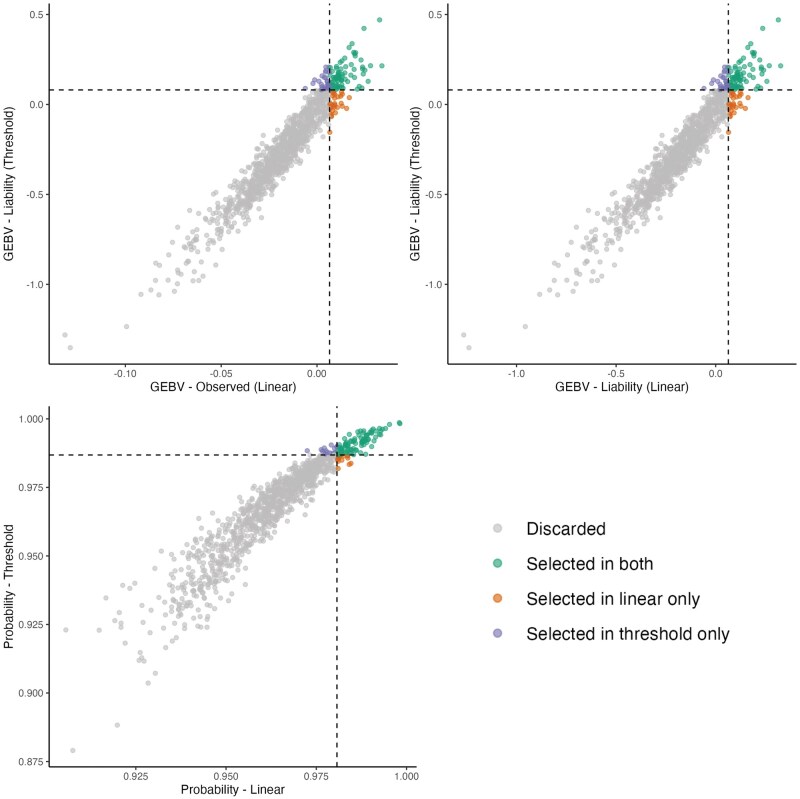
Commonly selected animals for the chamfer trait across models. Left: GEBVs from the linear model on the observed scale vs. GEBVs from the threshold model on the liability scale, considering only animals with more than 10 offspring. Right: GEBVs from the linear model on the liability scale vs. GEBVs from the threshold model on the liability scale. Animals are classified according to their selection status: not selected in either model, selected in both models, selected only in the linear model, or selected only in the threshold model.

A total of 1,050 sires with more than 10 phenotyped offspring were evaluated, with an average of 104 offspring per sire. Among the top 10% based on GEBVs from the linear and threshold models (105 animals), 74 sires were commonly selected by both models, while 31 were selected exclusively by one or the other. Based on the 10% selection threshold, 914 sires were discarded. The Spearman correlation among the top 10% animals was 0.95 for all the scales (Figure 6). Despite a moderate trait prevalence, the high agreement within the scales suggests a stability in animal ranking across models.

##### Loin

**Figure 7.**
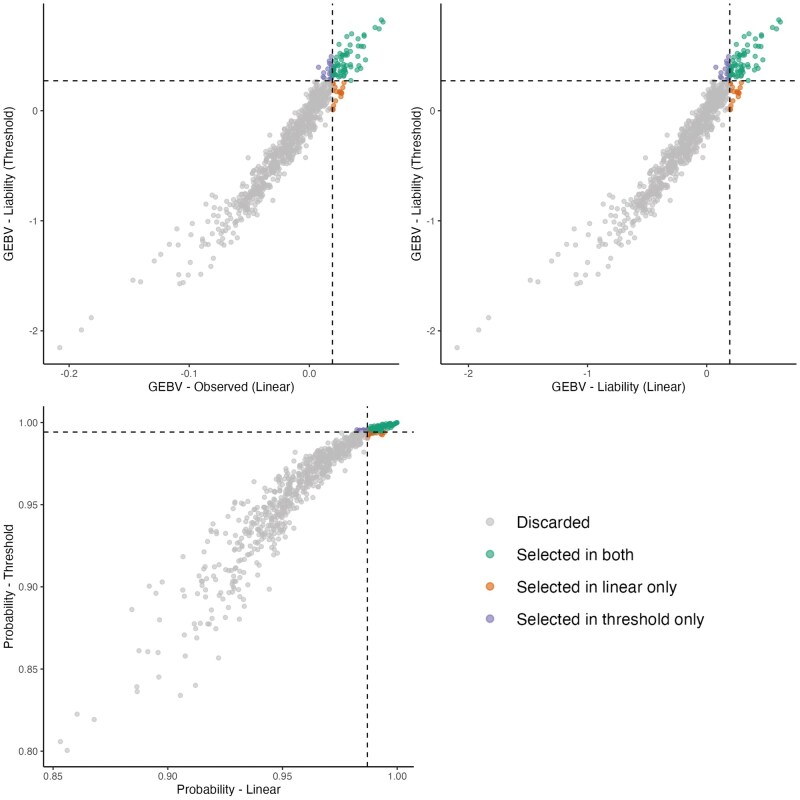
Commonly selected animals for the loin trait across models. Left: GEBVs from the linear model on the observed scale vs. GEBVs from the threshold model on the liability scale, considering only animals with more than 10 offspring. Right: GEBVs from the linear model on the liability scale vs. GEBVs from the threshold model on the liability scale. Animals are classified according to their selection status: not selected in either model, selected in both models, selected only in the linear model, or selected only in the threshold model.

A total of 804 sires with more than 10 phenotyped offspring were evaluated, with an average of 92 offspring per sire. Among the top 10% based on GEBVs from the linear and threshold models (81 animals), 65 sires were commonly selected by both models, while 16 were selected exclusively by one or the other. Based on the 10% selection threshold, 707 sires were discarded. The Spearman correlation among the top 10% animals was 0.97 for all the scales (Figure 7).

##### Hump

**Figure 8.**
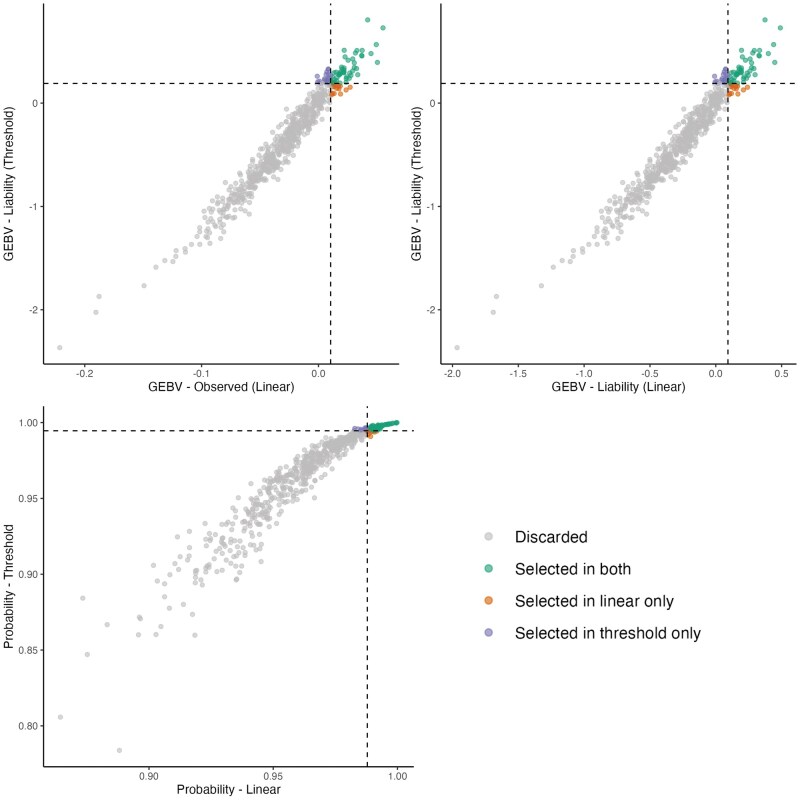
Commonly selected animals for the hump trait across models. Left: GEBVs from the linear model on the observed scale vs. GEBVs from the threshold model on the liability scale, considering only animals with more than 10 offspring. Right: GEBVs from the linear model on the liability scale vs. GEBVs from the threshold model on the liability scale. Animals are classified according to their selection status: not selected in either model, selected in both models, selected only in the linear model, or selected only in the threshold model.

A total of 637 sires with more than 10 phenotyped offspring were evaluated, with an average of 85 offspring per sire. Among the top 10% based on GEBVs from the linear and threshold models (64 animals), 47 sires were commonly selected by both models, while 17 were selected exclusively by one or the other. Based on the 10% selection threshold, 556 sires were discarded. The Spearman correlation among the top 10% animals was 0.97 for all the scales (Figure 8).

##### Jaw

**Figure 9.**
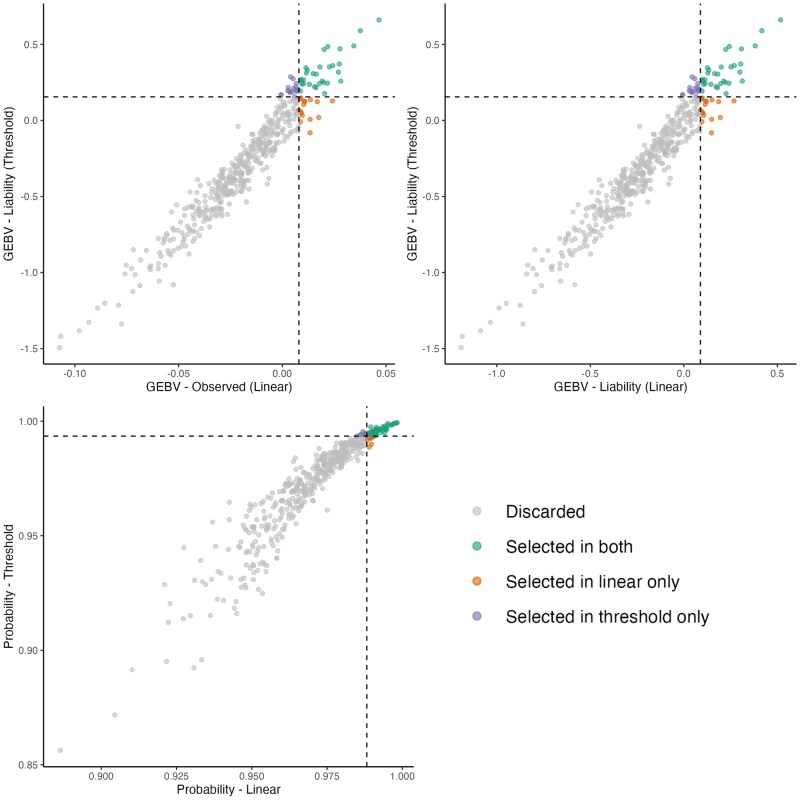
Commonly selected animals for the jaw trait across models. Left: GEBVs from the linear model on the observed scale vs. GEBVs from the threshold model on the liability scale, considering only animals with more than 10 offspring. Right: GEBVs from the linear model on the liability scale vs. GEBVs from the threshold model on the liability scale. Animals are classified according to their selection status: not selected in either model, selected in both models, selected only in the linear model, or selected only in the threshold model.

A total of 463 sires with more than 10 phenotyped offspring were evaluated, with an average of 69 offspring per sire. Among the top 10% based on GEBVs from the linear and threshold models (47 animals), 31 sires were commonly selected by both models, while 16 were selected exclusively by one or the other. Based on the 10% selection threshold, 400 sires were discarded. The Spearman correlation among the top 10% animals was 0.96 for all the scales (Figure 9).

##### Navel

**Figure 10.**
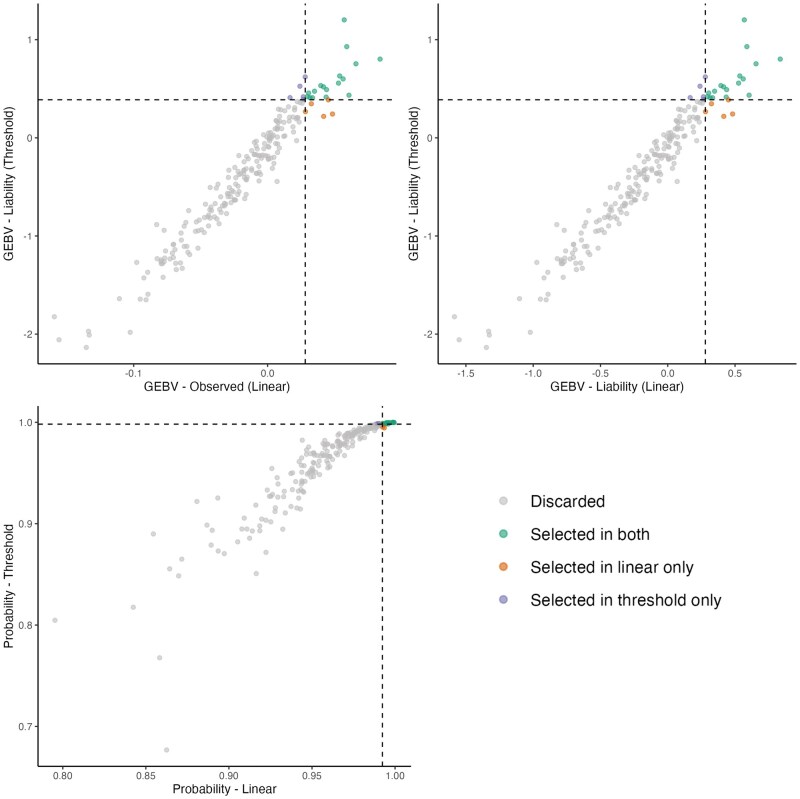
Commonly selected animals for the navel trait across models. Left: GEBVs from the linear model on the observed scale vs. GEBVs from the threshold model on the liability scale, considering only animals with more than 10 offspring. Right: GEBVs from the linear model on the liability scale vs. GEBVs from the threshold model on the liability scale. Animals are classified according to their selection status: not selected in either model, selected in both models, selected only in the linear model, or selected only in the threshold model.

A total of 219 sires with more than 10 phenotyped offspring were evaluated, with an average of 52 offspring per sire. Among the top 10% based on GEBVs from the linear and threshold models (22 animals), 17 sires were commonly selected by both models, while five were selected exclusively by one or the other. Based on the 10% selection threshold, 192 sires were discarded. The Spearman correlation among the top 10% animals was 0.97 for all the scales (Figure 10).

Although navel defects had a low prevalence and fewer total sires in the analysis, the correlation between models was high (0.97). The higher agreement in selected sires suggest that both models were able to identify similar outliers despite modeling differences. Still, the relatively small number of sires evaluated for this trait requires caution for interpretation.

### Genetic correlations among morphological defects

To explore the relationships among morphological defects in Nellore cattle, we calculated Pearson correlations between genomic estimated breeding values (GEBVs) obtained from both linear and threshold models. The goal was to evaluate whether traits exhibit shared genetic patterns or behave independently, which has implications for multiple-trait genetic evaluations and selection decisions.

**Figure 11.**
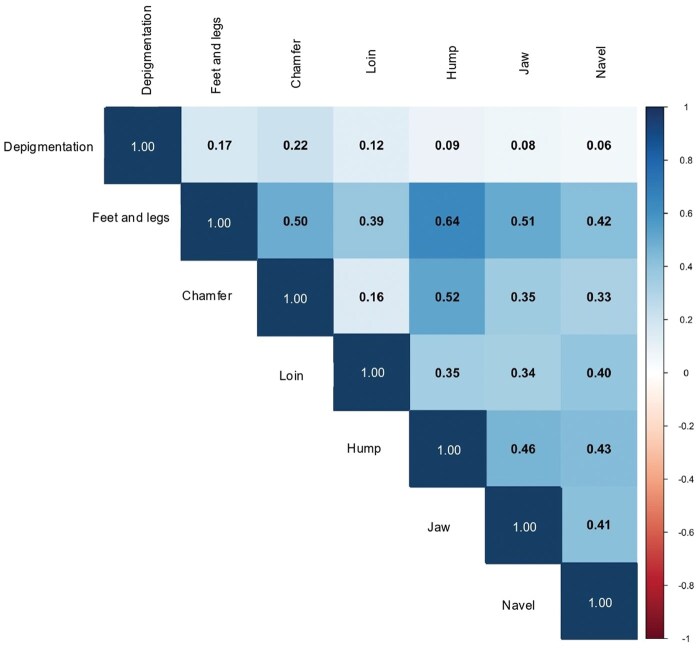
Pearson correlations from the GEBVs estimated using linear models (observed scale).

The Pearson correlations among traits on the observed scale (Figure 11) revealed several moderate to strong associations. For instance, feet and legs malformations showed moderate positive correlations with chamfer (0.50) and jaw defects (0.51), indicating possible shared genetic architecture related to conformation. Chamfer and hump also had a moderate correlation (0.52), which may be linked to head and upper-body morphological alignment. Loin was positively correlated with jaw (0.34) and feet and legs (0.39). Depigmentation showed weak correlations with other traits (all below 0.20), suggesting more distinct genetic control. These results indicate some clustering of skeletal structure traits (e.g., feet, jaw, chamfer), whereas traits like depigmentation may follow more independent genetic mechanisms.

**Figure 12.**
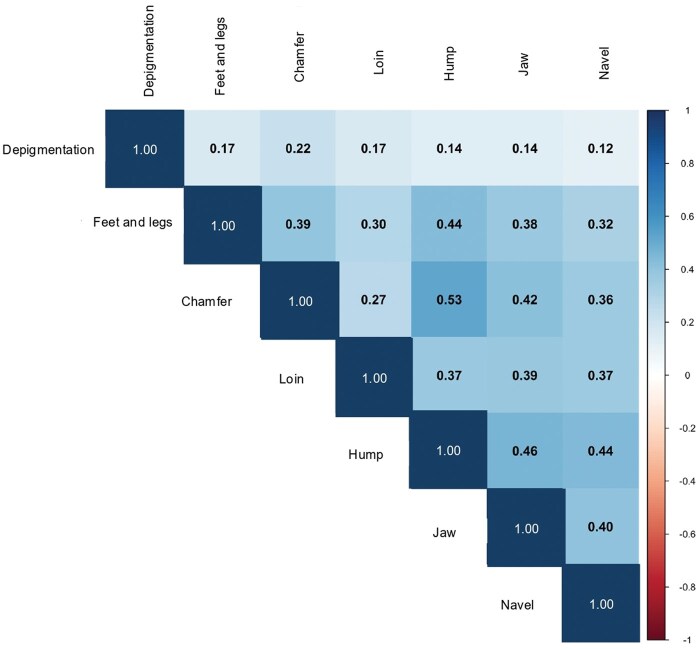
Pearson correlations from the GEBVs estimated using threshold models (liability scale).

Interestingly, the correlations on the liability scale, derived from threshold models, mostly preserved the direction and relative strength of associations seen in the linear model (Figure 12).

## Discussion

### Prevalence of morphological defects

Historically, the recording of morphological defects in Nellore breeding programs has been limited. The primary emphasis has often been on phenotypic selection against visibly abnormal animals through culling, rather than proactive genetic evaluation and selection (Silva et al., 2023; Diaz-Miranda et al., 2024). While this approach has contributed to reducing the frequency of some extreme phenotypes, it has not eliminated the defects. One contributing factor is the lack of standardized recording protocols and limited phenotypic data, particularly in earlier years of breeding programs (Figure 1).

Traditionally, evaluations in beef cattle have prioritized traits with direct economic value, such as growth, carcass quality, and navel structure, while subtler morphological anomalies have often been underreported (Souza et al., 2020). Recently, more comprehensive phenotyping protocols have been adopted, particularly within CEIP-certified programs, which now encourage a broader assessment of conformation and structural soundness. These programs recognize that improving the population’s genetics for such defects can reduce involuntary culling, and enhance productivity and overall animal welfare (Fernandes Júnior et al., 2022).

Depigmentation (6.18% prevalence; Figure 1), for instance, can result in sunburn and increased susceptibility to UV radiation (Gebremedhin et al., 2023). Similarly, even mild leg and feet malformations (7.44% prevalence; Figure 1) can impair locomotion, grazing efficiency, and overall productivity, which are particularly important in extensive systems like those commonly used in Brazil (Silva et al., 2023). Chamfer malformations (5.05% prevalence; Figure 1) may affect the respiratory system, potentially impairing heat dissipation and reducing productivity. Interestingly, Soares et al. (2023), when evaluating visual criteria including racial aspects such as chamfer, concluded that these traits can be beneficial for anticipating decision-making in selection processes. Loin defects (4.76% prevalence; Figure 1) can negatively impact carcass conformation and meat yield, while jaw malformation (4.23% prevalence; Figure 1) may reduce feed and water intake efficiency. Navel abnormalities (4.84% prevalence; Figure 1), a relatively more studied trait, can cause injuries to the prepuce in males, compromising reproductive performance (Araújo et al., 2010). In extensive systems, animals with excessively developed navels are more vulnerable to injuries, infections, and hernias (Herrmann et al., 2001).

Despite the clear impact of morphological defects on animal health, welfare, and productivity, comprehensive studies detailing their prevalence in Nellore cattle remain scarce. While some research addresses specific defects such as feet and leg malformations (e.g., Vargas et al., 2022) or genetic analyses of visual scores that may include aspects of conformation (e.g., Soares et al., 2023), there is a notable absence of broad, systematic investigations into the overall prevalence across a range of traits, particularly in large-scale commercial populations.

Our results show that over the years, both the number of recorded animals and the prevalence of these defects have increased (Figure 1a and c). This rise is likely due to improved data collection and growing awareness of these traits within breeding programs, as more farms seem to be contributing to data collection over the years (Figure 1b). Nevertheless, data collection for morphological defects in Nellore cattle remains a complex and evolving challenge. Variation in technician expertise, subjective assessments, and limited trait standardization can reduce recording consistency. More importantly, despite phenotypic culling, the continued presence (and in some cases increase) of these defects indicates that animals carrying the associated, undesirable alleles may still be used in breeding programs. Because these defects are polygenic, many animals may appear phenotypically normal while still carrying multiple small-effect alleles that contribute to the expression of the defect, allowing these alleles to persist unnoticed in the population. This highlights the limitations of relying solely on visual assessments and underscores the need for robust genetic management tools capable of identifying carrier animals. Integrating genomic information into breeding decisions is essential to avoid the inadvertent propagation of deleterious alleles and to support long-term improvements in structural soundness and animal welfare (Fernandes Júnior et al., 2022; Silva et al., 2023).

### Model comparison

In this study, we compared genetic parameter estimates and GEBVs obtained using linear and threshold models for the evaluation of binary traits related to morphological defects in Nellore cattle. The comparison focused on additive genetic variances, heritability estimates on both the liability and observed scales, and the agreement between GEBVs after transformation. As shown in Table 2, heritabilities estimated with the linear model were consistently lower than those obtained with the threshold model. This outcome is expected, as linear models assume normally distributed residuals and homogeneous residual variance, which are assumptions not fully met for binary traits (Ramirez-Valverde et al., 2001; Cappelloni et al., 2022). In contrast, threshold models account for the categorical nature of the data by modeling an underlying liability, which allows for more appropriate estimation of genetic variances in this context (Falconer and Mackay, 1996).

To enable direct comparisons, heritabilities from the threshold model were transformed to the observed scale using standard methods (Dempster and Lerner, 1950; Lee et al., 2011). After this transformation, heritability estimates obtained from both models became comparable. Although the threshold model tended to produce slightly higher numerical values, the differences were marginal. The 95% highest posterior density intervals overlapped for all traits, indicating a lack of strong posterior evidence for differences between the models. This supports the use of linear models as a practical alternative for estimating genetic parameters for binary traits.

In general, the genetic parameters estimated in this study were slightly lower than previously reported values in the literature for similar traits. For instance, Silva et al. (2023) used Bayesian inference in a two-trait linear-threshold animal model to jointly analyze feet and legs malformations (binary) and yearling weight (continuous) in Nellore cattle. They reported a posterior mean heritability of 0.18 for feet and legs malformations. In another study, Boligon et al. (2016) demonstrated that using a categorical “navel score” yields moderate to high heritability estimates (0.22 for weaning navel development score and 0.42 for yearling navel score). In a large dataset of over 85,000 Nellore animals, heritability estimates for navel score at yearling were 0.29 using a linear animal model and 0.42 with a threshold model; lower estimates were observed at weaning (0.16 linear; 0.22 threshold; Boligon et al., 2016). These results indicate that threshold models may better capture the genetic signal for categorical traits, particularly when phenotypic variation is more nuanced (Boligon et al., 2016; Campos et al., 2019).

One contributing factor to the lower heritability estimates identified in our study may be the use of binary phenotypic recording. While this method is practical for large-scale field data collection, it reduces the granularity of trait expression and may not fully capture variation in severity or expression thresholds. This limitation can lead to inflated residual variances and biased estimates of genetic parameters, particularly under linear model assumptions (Gianola, 1982; Varona et al., 1999). In the present study, all morphological defects were recorded directly as binary outcomes (0 = absence, 1 = presence), as no multi-category scoring system was used for these traits. Therefore, modeling the defects as binary traits reflects the original data structure and aligns with the way the phenotypes were collected.

Navel defects can be heterogeneous in nature and difficult to define precisely for recording purposes. However, when evaluated as a specific condition, such as umbilical hernia, high heritability has also been observed. In a large study of German Fleckvieh (Simmental) calves (*n* = 53,105), Herrmann et al. (2001) reported a prevalence of 1.8% and a heritability of 0.40 on the liability scale using a threshold model, confirming a strong genetic component for this condition. Traits such as hump development, while often considered breed-defining characteristics in *Bos taurus indicus*, lack specific heritability estimates in the literature. Nonetheless, these features are generally assumed to be heritable. Boligon et al. (2016) conducted a study in Nellore cattle and found that treating “breed characteristics” as a composite trait yielded heritability estimates of approximately 0.15 using both linear and threshold models. No other previous studies were found in the literature for the other traits evaluated in this study.

Figure 2 presents the distribution of GEBVs obtained using linear and threshold models across different scales. For all traits, the transformation of GEBVs from the linear model to the liability scale did not result in complete overlap with the distributions obtained from the threshold model. These findings reinforce the importance of selecting models appropriate to trait characteristics and interpreting GEBVs within the context of the trait’s prevalence and expression dynamics.

Beyond the statistical and methodological comparison of models, these traits have practical implications for cattle production systems. Morphological defects, although often expressed at relatively low prevalence, have important economic consequences. Involuntary culling for structural abnormalities and mobility issues has been associated with increased replacement costs, shortened productive lifespans, and reduced herd efficiency (Orpin and Esslemont, 2010; Kerslake et al., 2018). In beef and dairy populations, traits that influence costs related to longevity, culling, welfare, and functional soundness can be incorporated into breeding objectives and selection indices (Kluyts et al., 2004). Structural soundness traits such as lameness, which arises from foot and leg abnormalities and leads to impaired mobility, welfare concerns, and involuntary culling, have demonstrable economic impact (Boakari et al., 2022). This strengthens the rationale for genetically evaluating morphological defects that similarly affect locomotion, feeding efficiency, and overall functionality. The heritability estimates observed here, coupled with the welfare and productivity implications of these defects, indicate that including them in multi-trait selection indices is both feasible and advantageous. Incorporating these traits into selection decisions may reduce involuntary culling, increase longevity, and enhance the sustainability and profitability of Nellore breeding programs.

#### Transformation of GEBVs to probabilities

One of our goals in this study was to evaluate whether GEBVs for morphological defects could be expressed on the probability scale. The novel approach proposed by Hidalgo et al. (2024a, 2024b) enables a more intuitive interpretation of genetic merit by linking breeding values to the expected probability of expressing a defect, which is particularly relevant for binary traits. Although efficient for traits with higher prevalence, this approach showed lower consistency across scales for traits with prevalence below 5%. The second approach tested in this study, which was proposed by Padilla et al. (in press), produced high correlations even for traits with fewer cases, such as jaw (4.23%) and navel (4.84%). Therefore, GEBVs from both the linear and threshold models were converted to the probability scale using this second approach and compared using Spearman correlation coefficients and dispersion plots (Figure 3). High correlations were observed for all traits, indicating strong agreement between models and minimal re-ranking of individuals.

From a theoretical standpoint, the relationship between observed and liability-scale heritabilities is nonlinear and depends on trait prevalence; this nonlinearity underscores the limitations of interpreting genetic parameters solely on the observed scale and supports the use of the liability scale for statistical estimation as described by Gianola (1979). As originally proposed by Dempster and Lerner (1950), transforming GEBVs to the probability scale can enhance their utility for selection and decision-making, particularly by providing a more intuitive measure of the likelihood of trait expression. It is important to note, however, that the probability scale has inherent limitations. Because it is bounded between 0 and 1, both genetic and environmental variances may behave non-linearly across the range of predicted values, potentially obscuring additive genetic effects that are more accurately modeled on the liability scale (Dempster and Lerner, 1950).

#### Concordance of top-ranked animals between models

To assess the consistency of sire selection between models, we compared the top 10% of sires ranked by GEBVs after applying the transformation approach that yielded the highest correlations across scales (i.e., the approach proposed by de Oliveira Padilha et al., 2025). Only sires with at least 10 phenotyped offspring for the respective trait were included, ensuring rankings were based on reliable breeding value estimates.

These findings emphasize that model choice should be trait-specific. For traits with higher prevalence, both models performed similarly after transformation, but for low-prevalence traits, the threshold model may retain advantages in ranking stability. Applying the correct transformation equations allows greater flexibility in model choice, enabling breeding programs to balance computational efficiency, interpretability, and selection accuracy (Falconer and Mackay, 1996; Ramirez-Valverde et al., 2001).

### Genetic correlations among morphological defects

The Pearson correlations among GEBVs revealed trait-specific patterns of genetic association (Figure 11). Traits related to skeletal structure, such as feet and legs malformations, jaw defects, and chamfer malformation showed moderate positive correlations (ranging from 0.35 to 0.51), suggesting a shared genetic basis likely linked to overall conformation. Similar patterns have been reported in other structural traits, where genetic correlations reflect pleiotropy or linkage among genes affecting musculoskeletal development (Boligon et al., 2016; Souza et al., 2020). In contrast, depigmentation showed weak correlations with other traits (lower than 0.20), supporting its likely independence from structural traits. This aligns with previous findings indicating that pigmentation traits in cattle are influenced by distinct genetic pathways, often involving coat color or melanocyte function (e.g., Vargas et al., 2022; Gebremedhin et al., 2023). The consistency of correlation patterns across linear and threshold models reinforces the robustness of these relationships and supports the use of linear models for multiple-trait evaluations when model assumptions are carefully addressed (Koeck et al., 2010; Hidalgo et al., 2024a).

As more genotyped young animals become available in the DeltaGen population, future work should incorporate genomic prediction validation to directly compare the predictive performance of linear and threshold models. Future studies should also explore genetic correlations between morphological defects and production traits, which may help clarify possible indirect effects of selection and support the development of balanced breeding objectives.

## Conclusion

Our analysis revealed that morphological defects are present across the population with varying prevalence rates, ranging from 4.23% for jaw defects to 7.44% for feet and legs malformations, with temporal trends showing increased prevalence over time, likely attributable to improved phenotyping protocols and expanded participation in data collection rather than true genetic deterioration. The comparative analysis of linear and threshold models demonstrated that both approaches yielded statistically similar heritability estimates after appropriate scale transformation, with values indicating low heritability (0.03–0.12 on the observed scale). However, threshold models consistently produced slightly higher estimates and broader GEBV distributions, particularly for traits with lower prevalence. The conversion of GEBVs to the probability scale was most effective when applying the second transformation approach, which maintained high correlations even for low-prevalence traits. Finally, genetic correlations estimated through GEBV correlations revealed moderate positive associations among structurally-related traits (feet and legs malformations with chamfer and jaw defects showing correlations of 0.50–0.51). Depigmentation exhibited weak correlations with other defects (<0.20), suggesting distinct genetic control mechanisms and supporting the feasibility of independent selection strategies for different categories of morphological defects. These results support the integration of these traits into routine genetic evaluations with careful consideration of model choice based on trait-specific characteristics and provide a foundation for developing comprehensive breeding strategies that balance productivity gains with improved animal welfare and structural soundness.

## Supplementary Material

skaf438_Supplementary_Data

## Abbreviations:

AA: homozygous reference genotype
AB: heterozygous genotype
BB: homozygous alternate genotype
BLUPF90: best linear unbiased prediction, family of programs for genetic evaluations
CG: contemporary group
FAPESB: Fundação de Amparo à Pesquisa do Estado da Bahia
G: genomic relationship matrix
GEBV: genomic estimated breeding value
GIBBSF90+: Bayesian inference software (BLUPF90 family)
GWAS: genome-wide association study
H: relationship matrix combining pedigree and genomic information
HD: high density (illumina bovine HD array)
HPD: highest posterior density
I: identity matrix
MAF: minor allele frequency
MCMC: Markov chain Monte Carlo
QC: quality control
SNP: single nucleotide polymorphism
