# Supplementary material for: Comparison of linear and threshold models for genetic evaluation of morphological defects in Nellore cattle
Source: J Anim Sci. 2025 Dec 24;104:skaf438. doi: 10.1093/jas/skaf438 (PMC12863953; doi:10.1093/jas/skaf438)
Supplement: skaf438_Supplementary_Data [file skaf438_supplementary_data.zip › Supplementary Material_nomarked.docx]

**Supplementary Material**

S1. Definitions and minimum criteria used to classify each morphological defect present in the population.

| Trait | Definition / Minimum requirement for classification as “affected” |
| --- | --- |
| Feet and legs | Presence of visible deviations in limb alignment or hoof structure (e.g., valgus/varus deviation, hoof rotation, structural imbalance) that impair or are expected to impair locomotion. |
| Chamfer | Lateral deviation or asymmetry of the muzzle or nasal bone clearly detectable during visual inspection. |
| Hump | Displacement or irregular conformation of the thoracic hump, typically involving lateral deviation or asymmetrical development. |
| Loin | Irregularities such as visible depressions, asymmetry, or abnormal curvature in the lumbar region. |
| Jaw | Presence of mandibular abnormalities including prognathism (overshot jaw), brachygnathism (undershot jaw), or clear lateral deviation of the mandible. |
| Depigmentation | Partial or complete loss of pigmentation in specific anatomical regions, including the muzzle, periocular areas, tail switch, or localized body patches, as visually assessed by trained technicians. |
| Navel | Enlarged, elongated, or pendulous umbilical structure; or an abnormally short navel in males leading to partial exposure of the penis. |

S2.Number of contemporary groups excluded by each criterion

| Trait | Initial number of CG | Less than 10 animals | Variability | Disconnected | Final number of CG |
| --- | --- | --- | --- | --- | --- |
| Depigmentation | 57,354 | 35,401 | 16,322 | 2 | 5,629 |
| Feet and Legs malformations | 57,264 | 35,353 | 18,376 | 1 | 3,534 |
| Chamfer | 57,254 | 35,432 | 18,192 | 2 | 3,628 |
| Loin | 57,224 | 35,478 | 19,254 | 4 | 2,488 |
| Hump | 57,191 | 35,474 | 19,854 | 6 | 1,857 |
| Jaw | 57,179 | 35,505 | 20,471 | 2 | 1,201 |
| Navel | 57,157 | 35,509 | 21,191 | 1 | 456 |

S3. Iteration-adjusted computational time (hours per 100,000 iterations) for variance component estimation under linear and threshold models.

| Trait | Model | Hours per 100k iterations |
| --- | --- | --- |
| Depigmentation | Threshold | 397.39 h |
|  | Linear | 113.53 h |
| Chamfer | Threshold | 1181.94 h |
|  | Linear | 1119.56 h |
| Feet & Legs | Threshold | 732.73 h |
|  | Linear | 135.22 h |
| Hump | Threshold | 170.40 h |
|  | Linear | 106.15 h |
| Loin | Threshold | 121.37 h |
|  | Linear | 101.96 h |
| Jaw | Threshold | 131.43 h |
|  | Linear | 96.67 h |
| Navel | Threshold | 561.82 h |
|  | Linear | 129.27 h |

S4. Iteration-adjusted computational time (hours per 10,000 iterations) for GEBV estimation under linear and threshold models.

| Trait | Model | Hours per 10,000 iterations |
| --- | --- | --- |
| Depigmentation | Threshold | 32.71 h |
|  | Linear | 26.92 h |
| Chamfer | Threshold | 5.60 h |
|  | Linear | 4.32 h |
| Feet & Legs | Threshold | 9.92 h |
|  | Linear | 6.95 h |
| Hump | Threshold | 12.43 h |
|  | Linear | 5.65 h |
| Loin | Threshold | 6.05 h |
|  | Linear | 4.73 h |
| Jaw | Threshold | 6.52 h |
|  | Linear | 3.70 h |
| Navel | Threshold | 3.63 h |
|  | Linear | 2.90 h |

S5. Total number of sires for each trait

| Trait | Total number of Sires |
| --- | --- |
| Depigmentation | 4,285 |
| Feet and Legs malformations | 3,554 |
| Chamfer | 3,867 |
| Loin | 3,319 |
| Hump | 2,561 |
| Jaw | 2,526 |
| Navel | 1,016 |

S6. Variance components estimation using the Threshold and Linear Models for the binary traits

| Traits | $\boldsymbol{\sigma}_{\mathcal{a}}^{\boldsymbol{2}}$ | | $\boldsymbol{\sigma}_{\mathcal{e}}^{\boldsymbol{2}}$ | |
| --- | --- | --- | --- | --- |
|  | Linear | Threshold | Linear | Threshold |
| Depigmentation | 0.006 | 1.197 | 0.050 | 1.007 |
| Feet and Legs malformations | 0.003 | 0.304 | 0.062 | 1.011 |
| Chamfer | 0.001 | 0.226 | 0.046 | 1.010 |
| Loin | 0.003 | 0.603 | 0.042 | 1.008 |
| Hump | 0.003 | 0.552 | 0.049 | 1.009 |
| Jaw | 0.001 | 0.357 | 0.039 | 1.009 |
| Navel | 0.004 | 1.049 | 0.042 | 1.007 |

$\sigma_{\mathcal{a}}^{2}$: genetic variance; $\sigma_{\mathcal{e}}^{2}$residual variance

S7. Posterior Means of estimates from the Threshold Models

| Trait | Parameters | Mean | HPD Inteval (95%) | |
| --- | --- | --- | --- | --- |
| Chamfer | $\sigma_{\mathcal{a}}^{2}$ | 0.226 | 0.185 | 0.271 |
|  | $\sigma_{\mathcal{e}}^{2}$ | 1.010 | 0.999 | 1.021 |
|  | $h_{l}^{2}$ | 0.183 | 0.155 | 0.212 |
| Depigmentation | $\sigma_{\mathcal{a}}^{2}$ | 1.197 | 1.052 | 1.355 |
|  | $\sigma_{\mathcal{e}}^{2}$ | 1.007 | 0.997 | 1.016 |
|  | $h_{l}^{2}$ | 0.543 | 0.510 | 0.573 |
| Feet and Legs malformations | $\sigma_{\mathcal{a}}^{2}$ | 0.304 | 0.254 | 0.359 |
|  | $\sigma_{\mathcal{e}}^{2}$ | 1.011 | 1.000 | 1.023 |
|  | $h_{l}^{2}$ | 0.231 | 0.200 | 0.262 |
| Hump | $\sigma_{\mathcal{a}}^{2}$ | 0.552 | 0.414 | 0.707 |
|  | $\sigma_{\mathcal{e}}^{2}$ | 1.009 | 0.992 | 1.025 |
|  | $h_{l}^{2}$ | 0.352 | 0.293 | 0.414 |
| Jaw | $\sigma_{\mathcal{a}}^{2}$ | 0.357 | 0.250 | 0.473 |
|  | $\sigma_{\mathcal{e}}^{2}$ | 1.009 | 0.990 | 1.030 |
|  | $h_{l}^{2}$ | 0.260 | 0.200 | 0.321 |
| Loin | $\sigma_{\mathcal{a}}^{2}$ | 0.603 | 0.483 | 0.736 |
|  | $\sigma_{\mathcal{e}}^{2}$ | 1.008 | 0.993 | 1.021 |
|  | $h_{l}^{2}$ | 0.373 | 0.327 | 0.425 |
| Navel | $\sigma_{\mathcal{a}}^{2}$ | 1.049 | 0.607 | 1.559 |
|  | $\sigma_{\mathcal{e}}^{2}$ | 1.007 | 0.971 | 1.041 |
|  | $h_{l}^{2}$ | 0.504 | 0.386 | 0.616 |

$\sigma_{\mathcal{a}}^{2}$: genetic variance; $\sigma_{\mathcal{e}}^{2}$residual variance; h^2^: heritability; h^2^_l_: heritability on the liability scale; h^2^_o_: heritability on the observed scale

S8. Posterior Means of estimates from the Linear Models

| Trait | Parameters | Mean | HPD Interval (95%) | |
| --- | --- | --- | --- | --- |
| Depigmentation | $\sigma_{\mathcal{a}}^{2}$ | 0.006 | 0.006 | 0.007 |
|  | $\sigma_{\mathcal{e}}^{2}$ | 0.050 | 0.050 | 0.051 |
|  | $h_{o}^{2}$ | 0.109 | 0.101 | 0.118 |
| Feet and Legs malformations | $\sigma_{\mathcal{a}}^{2}$ | 0.003 | 0.002 | 0.003 |
| Chamfer | $\sigma_{\mathcal{a}}^{2}$ | 0.003 | 0.001 | 0.002 |
|  | $\sigma_{\mathcal{e}}^{2}$ | 0.001 | 0.046 | 0.047 |
|  | $h_{o}^{2}$ | 0.026 | 0.021 | 0.032 |
| Loin | $\sigma_{\mathcal{a}}^{2}$ | 0.003 | 0.002 | 0.003 |
|  | $\sigma_{\mathcal{e}}^{2}$ | 0.042 | 0.042 | 0.043 |
|  | $h_{o}^{2}$ | 0.057 | 0.048 | 0.066 |
| Hump | $\sigma_{\mathcal{a}}^{2}$ | 0.003 | 0.002 | 0.004 |
|  | $\sigma_{\mathcal{e}}^{2}$ | 0.049 | 0.049 | 0.050 |
|  | $h_{o}^{2}$ | 0.057 | 0.046 | 0.068 |
| Jaw | $\sigma_{\mathcal{a}}^{2}$ | 0.001 | 0.001 | 0.002 |
|  | $\sigma_{\mathcal{e}}^{2}$ | 0.039 | 0.038 | 0.040 |
|  | $h_{o}^{2}$ | 0.036 | 0.027 | 0.046 |
| Navel | $\sigma_{\mathcal{a}}^{2}$ | 0.004 | 0.002 | 0.005 |
|  | $\sigma_{\mathcal{e}}^{2}$ | 0.042 | 0.041 | 0.044 |
|  | $h_{o}^{2}$ | 0.084 | 0.053 | 0.113 |

$\sigma_{\mathcal{a}}^{2}$: genetic variance; $\sigma_{\mathcal{e}}^{2}$residual variance; h^2^: heritability; h^2^_l_: heritability on the liability scale; h^2^_o_: heritability on the observed scale

S9. Spearman correlation between breeding values from linear and threshold models using approach 1 (Hidalgo et al. 2024).

| Trait | Correlation | | |
| --- | --- | --- | --- |
|  | Observed, Liability | Liability 1, Liability 2 | Probability 3, Probability 4 |
| Depigmentation | 0.93 | 0.93 | 0.92 |
| Feet and legs malformation | 0.94 | 0.94 | 0.94 |
| Chamfer | 0.61 | 0.61 | 0.62 |
| Loin | 0.49 | 0.49 | 0.45 |
| Hump | 0.56 | 0.56 | 0.56 |
| Jaw | 0.48 | 0.48 | 0.38 |
| Navel | 0.32 | 0.32 | 0.23 |

Liability 1: from linear models (observed to liability); Liability 2: from threshold models (true liability); Probability 3: Liability 1 converted to probability scale; Probability 4: Liabilitity 2 to probability scale

S10. Spearman correlation between breeding values from linear and threshold models using approach 2 (Padilha et al. in press)

| Trait | Correlation | | |
| --- | --- | --- | --- |
|  | Observed, Liability | Liability 1, Liability 2 | Probability 3, Probability 4 |
| Depigmentation | 0.93 | 0.93 | 0.93 |
| Feet and legs malformations | 0.94 | 0.94 | 0.94 |
| Chamfer | 0.90 | 0.90 | 0.90 |
| Loin | 0.89 | 0.89 | 0.89 |
| Hump | 0.92 | 0.92 | 0.92 |
| Jaw | 0.91 | 0.91 | 0.91 |
| Navel | 0.92 | 0.92 | 0.92 |

Liability 1: from linear models (observed to liability); Liability 2: from threshold models (true liability); Probability 3: Liability 1 converted to probability scale; Probability 4: Liability 2 to probability scale


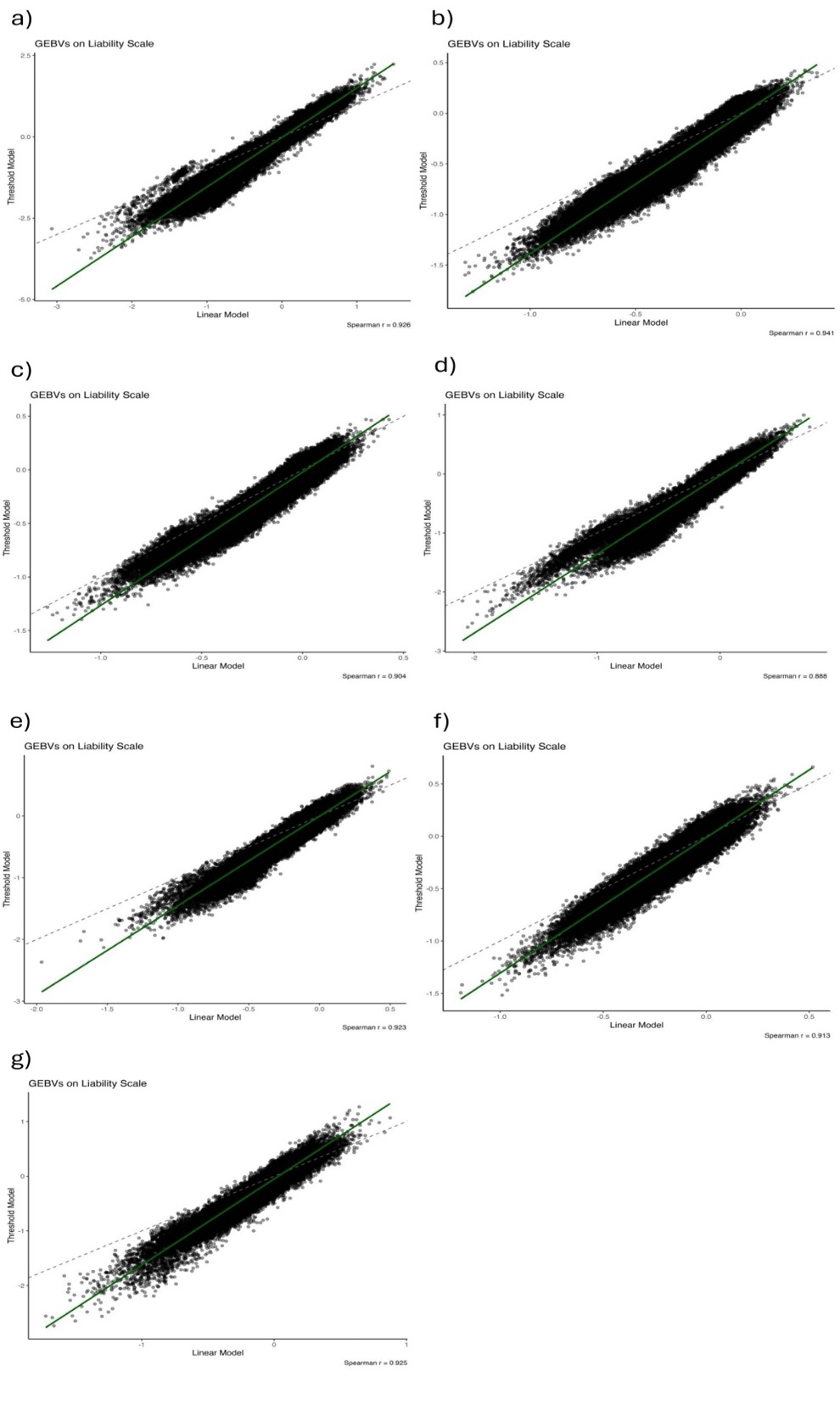


S11. Dispersion plots of liability-scaled GEBVs derived from both linear and threshold models for a) Depigmentation, b) Feet and legs malformations, c) Chamfer, d) Loin, e) Hump, f) Jaw, and g) Navel.
